# Supplementary material for: Learning curve and surgical outcome of robotic assisted colorectal surgery with ERAS program
Source: Sci Rep. 2022 Nov 29;12:20566. doi: 10.1038/s41598-022-24665-w (PMC9709162; doi:10.1038/s41598-022-24665-w)
Supplement: Supplementary file 1 — Supplementary Information 1. [file 41598_2022_24665_MOESM1_ESM.zip › LSC Raw data-Ñ[▒K/126/N_ñw╝╨░O▒Kñσ.pdf]

W55 / W55

臺中榮民總醫院
手術室護理記錄

[redacted]

基本資料

- 室別： 二醫手術室-31室，手術科別： CRS，手術類別： 常規，日期： 2021/05/06
- 進入時間： 1320 ； 麻醉開始時間： 1320 ； Time Out時間： 1500 ； 劃刀時間： 1501 ； 手術結束時間： 2030 送出時間： 2040 ；
- 麻醉方式： GE

  診斷：Malignant tumor of rectum PENDING OBSTRUCTION

  手術名稱： 1.Laparoscopic Anterior resection and anastomosis - malignant 2.Small intestine resection of small bowel (< 50 cm)3.Appendectomy4.

  手術局部用藥：NIL

  術前預防性抗生素：
  - 抗生素名稱 ： Veterin 1GM IVD/由麻醉護理師協助給予
  - 時間： 1400
  - 上次簽章: 蔡易軒

  , 追加預防性抗生素：
  - 抗生素名稱一： Veterin 1GM IVD/由麻醉護理師協助給予
  - 時間： 1800
  - 上次簽章: 黃鈺琇
- 手術醫師： 蔣鋒帆
- 麻醉醫師： VS 賴慧卿
-
  - 刷手護士A ： 彭馨慧，時間： 1320，接替刷手一： 黃鈺琇，時間： 1500，接替刷手二： 胡貞伶，時間： 1730
  - 流動護士A ： 蔡易軒，時間： 1320，接替流動一： 胡貞伶，時間： 1500，接替流動二： 顏育銹，時間： 1700
- 更多接替及流動護士： 接替刷手三： 謝昀安，時間： 2000，接替流動三： 黃鈺琇，時間： 1800

皮膚狀況

A-V Shunt： 無

術前： 完整

記錄者： 上次簽章: 蔡易軒

術後： 完整

記錄者： 黃鈺琇

手術準備

手術臥式 單一手術臥式

-
  - 手術臥式： 截石臥式
  - 使用附件： 溫毯 38 ℃
  - 皮膚準備： 10%Aq Betadine，Hibiscrub，2% Chlorhexidine
  - 電燒： 使用電燒: 電極片位置： 其它： 電燒墊，記錄者： 上次簽章: 蔡易軒

止血帶T： 無

植入物

- 植入物名稱/號碼/數量/部位：surgiwrap/0.02*130*200/骨盆腔

, 記錄者： 上次簽章: 黃鈺琇

標本送檢

- 細菌培養 無
- 細胞學 無
- 冰凍切片 無
- 病理標本

  組織名稱一：D-S colon

  ,

  組織名稱二：small intestine

  ,

  組織名稱三：appendix

  , 更多組織名稱
  - 組織名稱四：distal cutend
  - 組織名稱五：IMA root
- 截肢 無

標本交付 標本： 5 袋，標本： 0 盒，標本： 0 瓶，細胞學： 0 管，Culture Tube： 0 管，被交付醫師： R 古朋育，

- 記錄者： 上次簽章: 黃鈺琇
- 時間： 2000

傷口術後敷料

傷口敷料： 傷口敷料： 無

- 請於圖上標示部位：

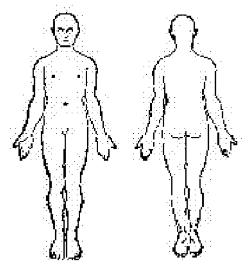

腹部直切傷口、腹腔鏡貳處切口

- 傷口敷料： 白紗+mepore覆蓋

導管

導管	天數	導管資訊	插入日期	插入時間	更換日期	拔除日期	拔除時間	拔除原因	預計拔除/更換	預計拔除/更換日期
A-line(1)	1	號碼:22 號; 部位:左手;	2021/05/06	1400						
Penrose Drain(1)	1	號碼:12 mm.; 部位:骨盆腔;	2021/05/06	1830						
Silicone Foley(1)	1	號碼:18 Fr.; 部位:膀胱; 註記:氣球打10CC;	2021/05/06	1508						
Sump NG Tube(1)	1	號碼:16 Fr.; 深度:60公分; 部位:胃;	2021/05/06	1400						

用物計數

- 手術中出血量： 150 c.c.
- 手術中尿量： 750 c.c.
-
  - 其它： 無

用物計數： 多切口同時縫合

- 1.器械： 正確，2.縫針： 正確，3.刀片： 正確，4.紗布： 正確，5.腹部墊： 正確，6.紗球： 未使用，7.PEANUTS ： 未使用，8.紗條： 未使用，9.絲帶： 未使用，10.針頭： 未使用，11.Vessel loop： 未使用，12.Boots： 未使用，13.Jelco： 未使用，14.棉條： 未使用，
  - 第一次：
    - 刷手護士 ： 胡貞伶
    - 流動護士： 上次簽章: 黃鈺琇
    - 時間： 1835

  ,
  - 第二次：
    - 刷手護士 ： 胡貞伶
    - 流動護士： 上次簽章: 黃鈺琇
    - 時間： 1910

  ,
  - 第三次：
    - 刷手護士 ： 胡貞伶
    - 流動護士： 上次簽章: 黃鈺琇
    - 時間： 1925

備註

備註： NIL

- 記錄者： 黃鈺琇 上次簽章: 胡貞伶
- 時間： 2040

填寫護理人員 黃鈺琇

正式摘要

W55 / W55

doctorRank:B
